# The role of MORC3 in silencing transposable elements in mouse embryonic stem cells

**DOI:** 10.1186/s13072-021-00420-9

**Published:** 2021-10-27

**Authors:** Varsha P. Desai, Jihed Chouaref, Haoyu Wu, William A. Pastor, Ryan L. Kan, Harald M. Oey, Zheng Li, Jamie Ho, Kelly K. D. Vonk, David San Leon Granado, Michael A. Christopher, Amander T. Clark, Steven E. Jacobsen, Lucia Daxinger

**Affiliations:** 1 Department of Molecular, Cellular and Developmental Biology, University of California, Los Angeles, Los Angeles, CA USA; 2 Department of Human Genetics, Leiden University Medical Center, Leiden, The Netherlands; 3 Department of Molecular Biology, Radboud University, Nijmegen, The Netherlands; 4 Present Address: Department of Biochemistry, McGill University, Montreal, QC Canada; 5 The University of Queensland Diamantina Institute, The University of Queensland, Woolloongabba, QLD 4102 Australia; 6 Present Address: Appia Bio, 6160 Bristol Parkway, Culver City, CA USA; 7 Eli & Edythe Broad Center of Regenerative Medicine & Stem Cell Research, University of California, Los Angeles, Los Angeles, CA USA; 8 Howard Hughes Medical Institute, University of California, Los Angeles, Los Angeles, CA USA; 9 The Rosalind & Morris Goodman Cancer Research Centre, McGill University, Montreal, QC Canada

**Keywords:** MORC3, Endogenous retroviruses, MommeD screen, Chromatin regulators, IAPs

## Abstract

**Background:**

Microrchidia proteins (MORCs) are involved in epigenetic gene silencing in a variety of eukaryotic organisms. Deletion of MORCs result in several developmental abnormalities and their dysregulation has been implicated in developmental disease and multiple cancers. Specifically, mammalian MORC3 mutations are associated with immune system defects and human cancers such as bladder, uterine, stomach, lung, and diffuse large B cell lymphomas. While previous studies have shown that MORC3 binds to H3K4me3 in vitro and overlaps with H3K4me3 ChIP-seq peaks in mouse embryonic stem cells, the mechanism by which MORC3 regulates gene expression is unknown.

**Results:**

In this study, we identified that mutation in *Morc3* results in a suppressor of variegation phenotype in a *Modifiers of murine metastable epialleles Dominant* (*MommeD*) screen. We also find that MORC3 functions as an epigenetic silencer of transposable elements (TEs) in mouse embryonic stem cells (mESCs). Loss of *Morc3* results in upregulation of TEs, specifically those belonging to the LTR class of retrotransposons also referred to as endogenous retroviruses (ERVs). Using ChIP-seq we found that MORC3, in addition to its known localization at H3K4me3 sites, also binds to ERVs, suggesting a direct role in regulating their expression. Previous studies have shown that these ERVs are marked by the repressive histone mark H3K9me3 which plays a key role in their silencing. However, we found that levels of H3K9me3 showed only minor losses in *Morc3* mutant mES cells. Instead, we found that loss of *Morc3* resulted in increased chromatin accessibility at ERVs as measured by ATAC-seq.

**Conclusions:**

Our results reveal MORC3 as a novel regulator of ERV silencing in mouse embryonic stem cells. The relatively minor changes of H3K9me3 in the *Morc3* mutant suggests that MORC3 acts mainly downstream of, or in a parallel pathway with, the TRIM28/SETDB1 complex that deposits H3K9me3 at these loci. The increased chromatin accessibility of ERVs in the *Morc3* mutant suggests that MORC3 may act at the level of chromatin compaction to effect TE silencing.

**Supplementary Information:**

The online version contains supplementary material available at 10.1186/s13072-021-00420-9.

## Background

Epigenetic features such as histone modifications, DNA methylation (5-methylcytosine or 5mC) and chromatin accessibility play central roles in modulating transcriptional output. For example, repressed regions are enriched in histone marks such as H3K9me3 and H3K27me3, have high 5mC and are less accessible, whereas active regions are associated with histone marks such as H3K4me3 and H3-acetylation, have reduced 5mC and are relatively more accessible. Several protein factors are responsible for regulating and translating these epigenetic marks into specific gene expression signals. A recently discovered family of proteins called MORCs have been shown to play a key role in the regulation of the epigenome in plants, worms and mammals [1–3]. Deletion of MORCs results in severe developmental phenotypes including germ line sterility, immune system defects and neural disorders [1–4]. Moreover, MORCs have been implicated in tumor suppression and in many cancers [5–11]. Given these dramatic phenotypes, understanding the molecular mechanisms by which MORCs regulate gene expression is of utmost importance.

Mammalian MORCs are comprised of four genes (MORC1, MORC2, MORC3 and MORC4), and emerging evidence suggests a key role of MORCs in regulating transposable element (TE) expression in both mice and humans [12–19]. For example, deletion of MORC1 causes deficient establishment of DNA methylation at transposons during mouse male germline development resulting in male-specific infertility [19]. Similarly, MORC2 has been implicated in silencing young retrotransposons as an accessory member of the human silencing hub (HUSH) complex and has been shown to be involved in depositing H3K9me3 and locally compacting chromatin [15, 16].

Biochemical studies have shown that MORC1 and MORC2 do not bind histone marks [20]. In contrast, MORC3 and MORC4 have a functional CW domain with a preference for binding methylated H3K4, with the strongest affinity to H3K4me3 [6, 20–23]. Previous studies have shown that MORC3 localizes to promoters of actively transcribing genes in vivo but its function at these loci remains unknown [21]. These observations raise an important question as to whether MORC3 acts as a repressor in vivo like other members of the MORC family, or whether MORC3 may act as an activator given its association with H3K4me3.

In this study, we identified MORC3 as a potential epigenetic silencer through a *Modifiers of murine metastable epialleles Dominant* (*MommeD*) screen. Furthermore, we discovered that loss of MORC3 in mESCs causes a global upregulation of ERVs, specifically young intracisternal A particles (IAPs). Furthermore, these ERVs are marked by MORC3 occupancy in wild type (WT) mESCs suggesting a direct role of MORC3 in regulating their expression. Loss of MORC3 resulted in only a minor reduction in the levels of the repressive histone mark H3K9me3 at a subset of upregulated ERVs, but instead led to substantial decompaction of chromatin. These results show that despite its ability to bind H3K4me3 at promoters, MORC3 also functions as a repressor of ERVs, where its activity is associated with chromatin compaction.

## Results

### A screen for epigenetic modifiers identifies an allele of *Morc3* as a suppressor of variegation

**Fig. 1:**
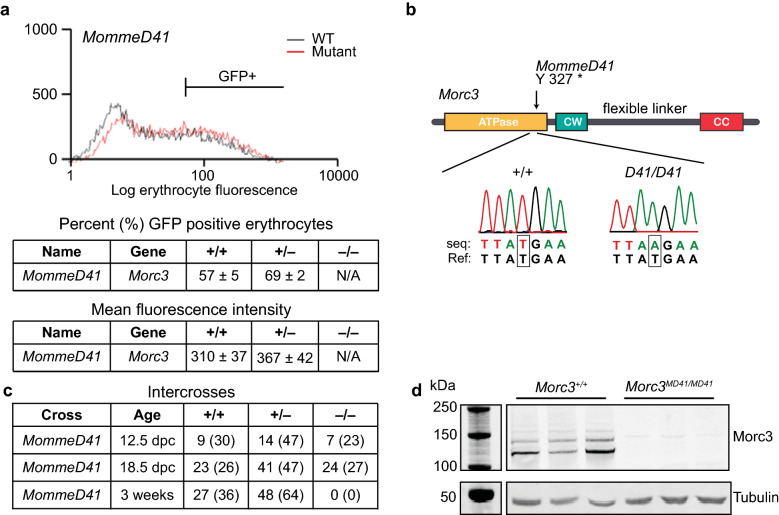
Identification of a *Morc3* allele from the *MommeD* screen. **a** FACS analysis and quantification of 10,000 erythrocytes from 3-week-old mice. Representative GFP profiles for *MommeD41* WT and *MommeD41* heterozygous mice (littermates) are shown. The *MommeD41* allele demonstrates a higher percentage of erythrocytes expressing GFP and an increased mean fluorescence of expressing cells compared to WTs. Red line represents *MommeD41* and black line represents WT. **b** Sanger sequencing trace of WT and *Morc3*^*MD41/ MD41*^ demonstrating a T to A transversion. **c** Timed matings and intercrosses show the number of embryos/mice observed (and in brackets the percentage) at the indicated timepoints. **d** Western blot validates the loss of MORC3 (~ 110 kDa) in *Morc3*^*MD41/MD41*^ mESCs (*n* = 3, biological replicates). Tubulin (~ 50 kDa) is shown as a loading control. The higher (> 110 kDa) bands may represent sumoylated forms of MORC3, as previously shown by Mimura et al. [54]

We previously reported a chemical mutagenesis based *MommeD screen* [24, 25]. This screen uses a mouse line (*Line3*) that is homozygous for a multicopy GFP transgene expressed in a variegated manner in 55% of red blood cells. *MommeD* mutants are assigned a *Suppressor of variegation* (*Su(var)*) or *Enhancer of variegation* (*E(var)*) phenotype based on increased or decreased percentage of red blood cells expressing GFP, respectively. The *MommeD41* allele was identified as a *Su(var),* because it showed a greater than 10% increase in the proportion of erythrocytes expressing GFP (Fig. 1a, Additional file 1: Figure S1). The flow cytometry read-out was used to follow the mutation by backcrossing for multiple generations before performing genetic linkage analyses and whole exome sequencing. Using the Illumina GoldenGate SNP genotyping assay, we identified a 26 Mb interval on chromosome 16 likely containing the *MommeD41* allele (Additional file 2: Table S1). To identify the underlying mutation, whole exome sequencing of DNA from a *MommeD41* heterozygote was carried out and variants were called within the identified linked interval. An exonic T to A mutation that results in a premature stop codon at Tyr327 was identified in the *Morc3* gene (Fig. 1b, Additional file 3: Table S2), and we, therefore, designated the *MommeD41* allele as *Morc3*^*MD41*^. Heterozygous *Morc3*^*MD41*^ mice were observed at expected ratios at weaning (Fig. 1c). To determine the viability of homozygotes, we performed heterozygous intercrosses and dissected and genotyped embryos at different stages of development. Viable homozygotes at the expected Mendelian ratios and without gross abnormalities were recovered up to E18.5, consistent with previous observations for a null *Morc3* allele showing that homozygous mice are initially normal but die at birth [26].

### Endogenous retroviruses are overexpressed in the absence of MORC3

Because of the perinatal lethality of *Morc3*^*MD41*^ homozygotes we derived mESC lines to study the role of MORC3 in gene regulation. One set of mESC lines were derived from *Morc3*^*MD41/MD41*^ and WT FVB preimplantation embryos and maintained in the presence of 2i and serum (Additional file 1: Figure S2). Another set of mESC lines were generated through CRISPR-Cas9 mediated deletion of exon#2 of the *Morc3* gene in V6.5 mESCs and was maintained in serum (Additional file 1: Figure S3). A control line was generated, where a plasmid expressing GFP was transfected instead of the CRISPR-Cas9 cassette, and this line is referred to as WT in the analysis below. All the mESC lines showed a morphology characteristic of the respective culturing regime and western blot analysis demonstrated loss of MORC3 protein in all the mutant lines (Fig. 1d and Additional file 1: Figures S2, S3).

**Fig. 2:**
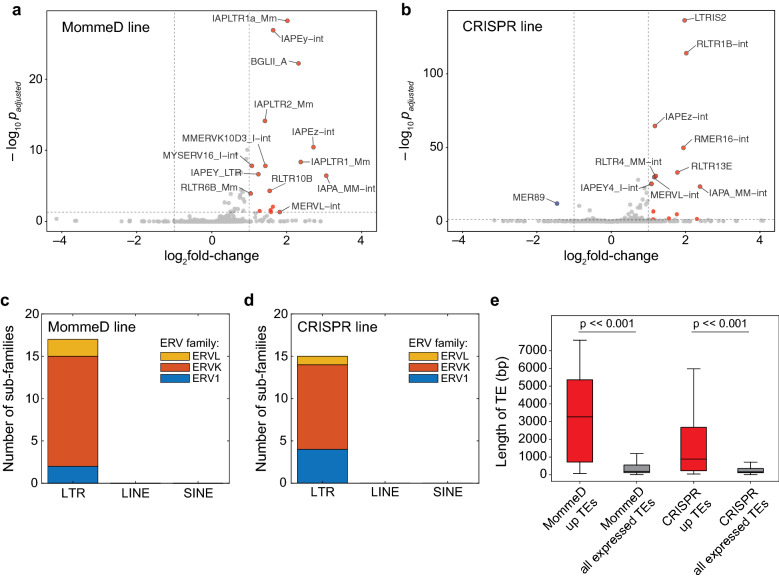
Loss of MORC3 results in upregulation of TEs. **a**, **b** Volcano plot showing log_2_fold change in TE expression between WT and *Morc3*^*MD41/MD41*^ (MommeD line,** a**), and between WT and *Morc3*^*–/–*^ (CRISPR line, **b**). TE expression of three replicates were measured and log_2_fold change >|1| and *p*(adj)-value < 0.05 was considered significant. Reads were allowed to multimap and a published TE transcripts pipeline was used to determine differentially expressed TEs [27]. **c**, **d** Barplot showing the distribution of upregulated TEs in the MommeD line (**c**) and the CRISPR line (**d**). **e** Barplots comparing the length of TEs that are upregulated in the MORC3 mutant lines with the length of all expressed TEs. Mann–Whitney *U* test was used to test for significance (*p*-value = 8.8e−73 in the MommeD line, *p*-value = 6.8e−39 in the CRISPR line)

To investigate the role of MORC3 in regulating gene expression, we performed RNA-seq in WT FVB, *Morc3*^*MD41/MD41*^, WT GFP and *Morc3*^*–/–*^ lines. While there were few expression changes in protein coding genes in common between the two *Morc3* mutant lines, we observed an upregulation of TEs, specifically at ERVs, which is consistent with the known role of other MORC family members in silencing repetitive elements [15–19]. We observed 17 upregulated ERV sub-families in the MommeD line and 14 upregulated ERV sub-families in the CRISPR line (Fig. 2a, b). These upregulated sub-families were identified by allowing reads to multimap to the genome and then using the TEtranscripts pipeline to identify upregulated sub-families [27]. In both lines, ERVK elements represented the largest family among the significantly upregulated ERVs (Fig. 2c, d).

We next asked how expression of individual TE insertions changes in the absence of MORC3 by analyzing uniquely mapped reads. We identified 260 upregulated TE insertions in the MommeD line and 175 upregulated TE insertions in the CRISPR line. Interestingly, we found that the average size of the TE insertions upregulated were significantly longer in both the MommeD and the CRISPR line suggesting that MORC3 is involved in silencing longer and more intact TEs (Fig. 2e). It is important to note that utilizing only uniquely mapped reads grossly undercounts the upregulated TEs, because many younger and repetitive TEs are challenging to map uniquely.

### Morc3 localizes to both gene promoters and endogenous retroviruses

**Fig. 3:**
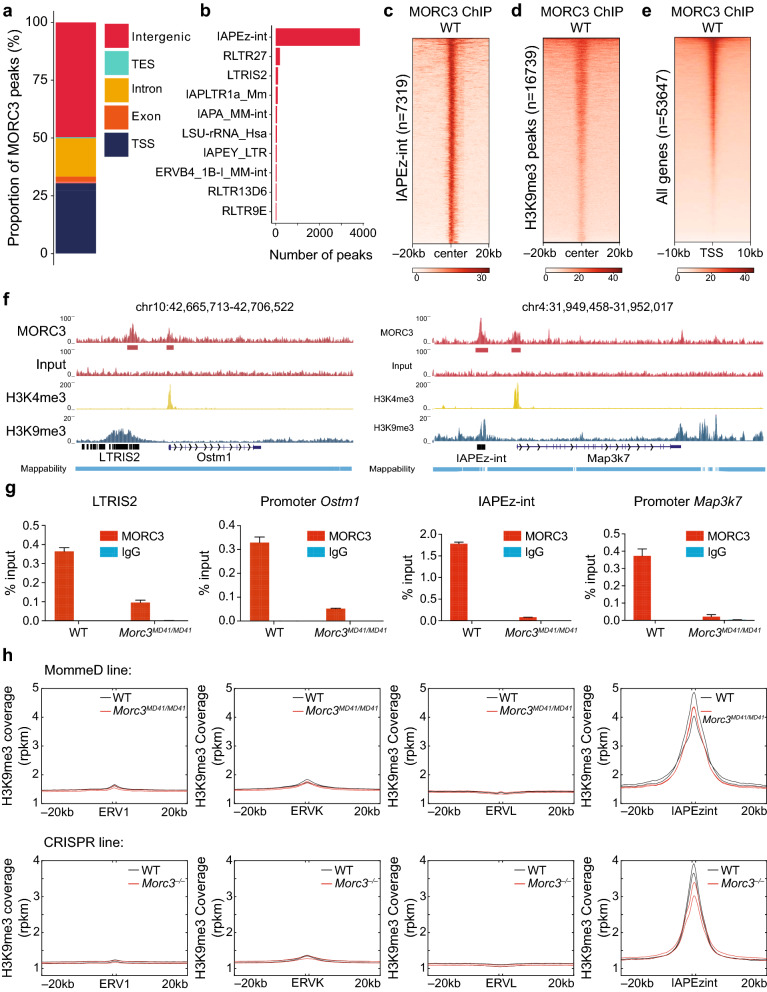
MORC3 localizes at transcription start sites and at ERVs. **a** Distribution of 9120 MORC3 peaks over genomic features in WT V6.5 mESCs (*n* = 4 replicates). **b** Distribution of MORC3 peaks localized in intergenic regions. **c** MORC3 enrichment at H3K9me3 marked regions. **d** MORC3 enrichment at promoters of all mouse genes. **e** Representative genome browser track at the *Ostm1* and *Map3k7* loci showing MORC3 peaks at promoters as well as at upstream ERVs. **f** MORC3 ChIP-qPCR at the *Ostm1* promoter and its nearby ERV (LTRIS2) and at the *Map3k7* promoter and its nearby ERV (IAPEz-int). **g** Metaplot showing H3K9me3 coverage (rpkm) at ERV1, ERVK, ERVL, IAPEzint and 20 kb flanking regions

Previous studies have shown that MORC3 binds to the histone mark H3K4me3 in vitro, and localizes to sites of H3K4me3 in vivo at promoters of actively transcribing genes [6, 20, 21, 23]. However, the identification of a *Morc3 Su(var)* allele from the MommeD screen together with our transcriptomics data suggested a role of MORC3 in epigenetic silencing. To reconcile these contrasting observations, we quantified the binding of MORC3 in WT V6.5 mESCs using ChIP-seq and identified 9119 peaks of which 2773 peaks (~ 30%) overlapped with transcription start sites (TSSs) and 4526 peaks (~ 50%) overlapped with distal intergenic regions (Fig. 3a and Additional file 4: Table S3). MORC3 peaks located in distal intergenic regions corresponded to TEs, and a majority of these peaks overlapped with the IAPEz-int subfamily (~ 85% of the Morc3 binding sites in intergenic regions, Fig. 3b, c). As an important control, we found that the enrichment of MORC3 at the IAPEz-int subfamily observed in wild type mESCs was lost in *Morc3*^*–/–*^ mESCs demonstrating the specificity of MORC3 binding at these repetitive elements (Additional file 1: Figure S4). Moreover, consistent with our RNA-seq results, we found that MORC3 localizes to longer repetitive elements in most of the MORC3-bound subfamilies (Additional file 1: Figure S5).

These results suggest that MORC3 can localize to two very different chromatin states: at active gene promoters marked by H3K4me3, and at ERVs that are often silenced through the deposition of the repressive histone mark H3K9me3. Indeed, when we performed H3K9me3 ChIP-seq in WT mESCs, we found that MORC3 was enriched over all H3K9me3 peaks (n = 16,739) (Fig. 3d, Additional file 5: Table S4). Similar coverage analysis over all gene promoters showed that MORC3 was also enriched at transcriptional start sites (TSS, Fig. 3e). Notably, MORC3 bound TSS did not show any enrichment of H3K9me3, and MORC3 bound at H3K9me3 loci did not show any enrichment of H3K4me3 (Additional file 1: Figure S6), making it unlikely that MORC3 recognizes a combination of H3K4me3 and H3K9me3. Representative genome browser shots at *Ostm1* and *Map3k7* showed the presence of MORC3 at both a gene promoter (marked by H3K4me3) and at an upstream ERV (marked by H3K9me3, Fig. 3f). We validated these observations by performing ChIP-qPCR in WT and *Morc3*^*MD41M/D41*^ mESCs at multiple genomic loci. For example, MORC3 was enriched at the TSS of *Ostm1* and the upstream LTRIS2 element as well as at TSS of *Map3k7* and the upstream IAPEz-int element (Fig. 3g, and additional ChIP-qPCR shown in Additional file 1: Figure S7).

Together with the transcriptomic analysis, these results suggest that IAP elements are the main target of MORC3, showing the strongest upregulation and the strongest association with MORC3 peaks. In contrast, another member of the MORC family of proteins, MORC2A, has been shown to be enriched primarily at LINE elements (Additional file 1: Figure S8) suggesting that different MORCs may be involved in regulating different types of repetitive elements [18].

### Loss of MORC3 results in a minor reduction of H3K9me3 and gain in chromatin accessibility at ERVs

Endogenous retroviruses have previously been shown to be silenced through the deposition of the repressive histone mark H3K9me3 [28, 29]. As MORC3 binds to ERVs and loss of MORC3 results in ERV upregulation, we performed H3K9me3 ChIP-seq in both the MommeD and the CRISPR *Morc3* mutant lines to investigate if the loss of MORC3 influenced the deposition of H3K9me3 at these elements. We found that *Morc3* mutant cell lines showed a very minor reduction of H3K9me3 over ERVs genome-wide (Fig. 3h) with many ERV subfamilies showing no reduction, and others showing a minor reduction (Additional file 1: Figure S9). The minor loss of H3K9me3 and the fact that many ERVs directly bound by MORC3 did not display a significant loss of H3K9me3 suggests that the upregulation of ERVs upon the loss of MORC3 is mostly independent of the deposition of H3K9me3.

**Fig. 4:**
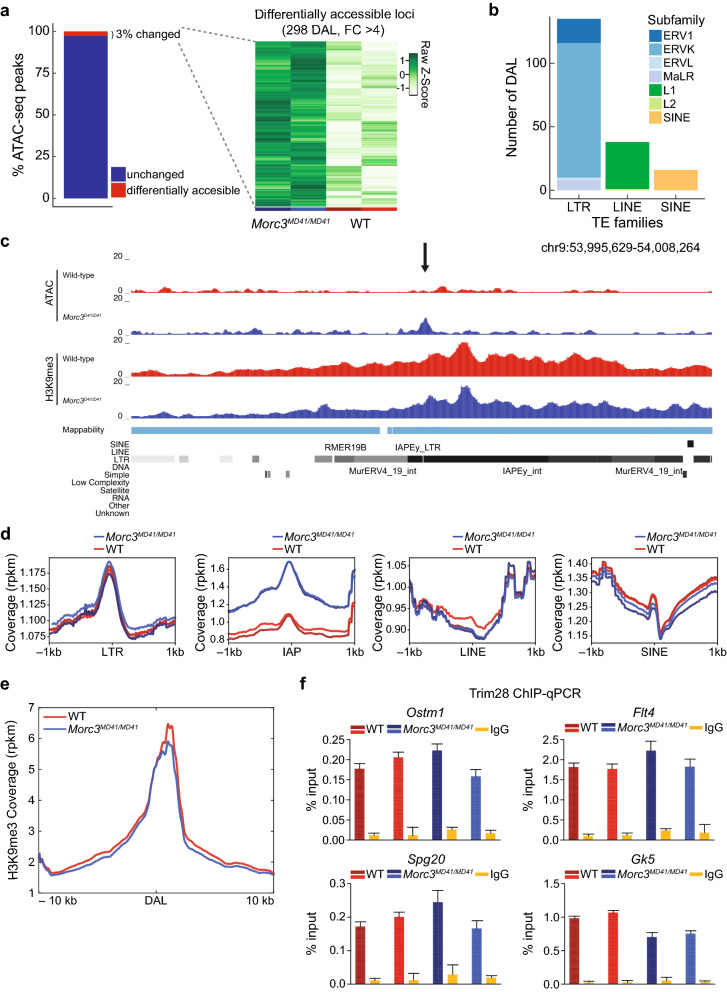
MORC3 regulates chromatin accessibility at ERVs. **a** Percentage of the total ATAC peaks (n = 11,000) that show increased chromatin accessibility (left panel) and heatmap showing the difference in chromatin accessibility between *Morc3*^*MD41/MD41*^ and the WT (right panel, *n* = 2 biological replicates). Each row corresponds to chromatin accessibility of a single locus. **b** Barplot showing the annotation of the 298 differentially accessible loci (DAL). **c** Representative genome browser tracks showing a change in accessibility in *Morc3*^*MD41/MD41*^ occurring at an LTR elements (RepeatMasker track) covered with H3K9me3. The DAL is shown by the black arrow. **d** Metaplot showing the ATAC-seq coverage at LTR, IAP, LINE and SINE in WT (red) and *Morc3*^*MD41/MD41*^ (blue) with 1 kb flanking region. Replicates are shown in a different shade of the same color. **e** Metaplot showing the average H3K9me3 read coverage at all DAL. **f** Trim28 ChIP-qPCR at DAL in WT (red) and *Morc3*^*MD41/MD41*^ (blue)

As other MORC proteins have been implicated in chromatin compaction, we tested whether an alternative explanation for the increased expression of ERVs in *Morc3* mutant cell line might be differences in chromatin accessibility. To this end, we performed ATAC-seq in two WT and two *Morc3*^*MD41/MD41*^ mESC lines. Our ATAC-seq data showed a typical enrichment of ATAC-seq reads around the TSS of genes in both WT and *Morc3*^*MD41/MD41*^ cell lines (Additional file 1: Figure S10a). We identified 298 peaks that showed significantly higher chromatin accessibility in *Morc3*^*MD41/MD41*^ cell lines (fold-change > 4, *p* value < 0.05), representing 3% of the total number of accessible peaks in mESCs (Fig. 4a). These differentially accessible loci (DAL) occurred primarily over TEs and particularly over the LTR class of TEs (135/298, Fig. 4b). Specifically, we observed increased chromatin accessibility at IAP elements in the *Morc3*^*MD41/MD41*^ mutant lines, whereas other LTR-containing ERVs, and the non-LTR containing LINE and SINE elements remained unchanged (Fig. 4c, d). Interestingly, we found that while there was a slight increase in accessibility at MORC3 peaks in the absence of MORC3, this increase predominantly arose from the gain in accessibility at IAPEz-ints as there was no measured changed in accessibility at MORC3-bound promoters in the absence of MORC3 (Additional file 1: Figure S11).

Since IAPs are silenced by the deposition of H3K9me3 via the TRIM28/SETDB1 pathway [28, 29], we specifically examined the impact of MORC3 loss on the deposition of H3K9me3 at these DAL. Consistent with our global analysis of TEs we observed similar levels of H3K9me3 in WT and *Morc3*^*MD41/MD41*^ cell lines at DAL (Fig. 4e, Additional file 1: Figure S10b). We also tested whether MORC3 loss had any impact on the presence of TRIM28 at these loci by performing TRIM28 ChIP-qPCR at DAL and other control loci in WT and *Morc3*^*MD41/MD41*^ mESCs. We found that using publicly available TRIM28 ChIP-seq data in mESCs that TRIM28 was enriched at DAL (Additional file 1: Figure S10c), but there were no differences in TRIM28 enrichment between WT and *Morc3*^*MD41/MD41*^ mESCs (Fig. 4f and Additional file 1: Figure S12). These results indicate that TRIM28 localizes at the DAL irrespective of the presence of MORC3.

## Discussion

In this study, we identified MORC3 as a novel silencer of ERV expression in mESCs, specifically IAPs. IAPs represent one of the youngest and most active families of mobile genetics elements in mice [28–31]. Initiation of IAP silencing is known to occur through their recognition by Krüppel-associated box domain-zinc finger proteins (KRAB-ZFPs) [32], which in turn recruit TRIM28 and SETDB1 to methylate H3K9 and repress these loci [28, 29]. We found that in the absence of MORC3, the localization of TRIM28 was unaffected and the levels of H3K9me3 were also largely unchanged suggesting that MORC3 acts downstream of, or in a parallel pathway to, the TRIM28/SETDB1 complex. These results share similarity with those of another member of the MORC family, MORC2A, which has been shown to repress LINE elements, especially evolutionarily young L1s, in cooperation with the HUSH complex [17, 18, 33]. L1 loci regulated by MORC2A are also repressed by TRIM28/SETDB1 such that both MORC2A/HUSH and TRIM28/SETDB1 are required for complete transcription repression. Like MORC3, loss of MORC2A results only in a minor loss of H3K9me3 [17, 18, 33]. Therefore, it is possible that the MORC family of proteins have evolved to specifically silence different classes of TEs in a pathway parallel to the TRIM28/SETDB1 pathway. As young TEs are more prone to erroneous activation, the role of MORCs could be particularly important at such metastable loci.

We found that MORC3 may regulate TE expression via chromatin compaction as we observed that the absence of MORC3 resulted in a gain of chromatin accessibility at IAPs. These findings are consistent with previous studies that have shown that several members of the MORC family of proteins in plants, mammals, and worms are important factors in regulating chromatin accessibility. For example, deletion of MORCs in *A. thaliana* results in decompacted chromocenters and expression of normally DNA methylated and repressed sites in the genome [34]. In mouse primordial germ cells, MORC1 was shown to be required for silencing of IAP elements, and *Morc1* mutant cells showed an increase in chromatin accessibility at repressed sites [19]. In humans, MORC2 has also been shown to play a role in regulating chromatin accessibility at heterochromatic loci that are enriched in zinc finger proteins [15]. In addition, *C. elegans* MORC1 mutants show chromatin decompaction in vivo, and in vitro* C. elegans* MORC1 is able to induce efficient compaction of naked DNA or chromatin [35]. While the role of MORC3 in chromatin compaction is suggested in vivo, future studies will be necessary to understand the exact mechanism by which MORC3 drives this compaction. Another intriguing question is how MORC3 is recruited to repressed ERVs. We found, consistent with a previous study, that MORC3 co-localized with H3K4me3 at gene promoters, and structural and biochemical analysis has shown that the MORC3 CW domain binds to H3K4me3 [6, 20, 21, 23]. ERVs, however, are enriched in the repressive H3K9me3 mark, and further investigation is required to determine how MORC3 is recruited to these sites.

## Methods

### MommeD line

#### Mouse strains and timed matings

Mice carrying the *MommeD41* mutation were produced on the FVB/NJ background homozygous for the Line3 GFP transgene, as described previously for other *MommeD* mutations [25, 36–39]. Maintenance of the *MommeD41* allele was carried out on the Line3 background. For timed matings heterozygous males were set up with heterozygous females and the detection of a vaginal plug was counted as 0.5 dpc. Genotyping was carried out using genomic DNA extracted from embryonic tissue.

#### Flow cytometry

One drop of tail blood of 3 week old mice was collected FACSFlow Sheath Fluid (BD Biosciences) and flow cytometry analysis was carried out and analyzed on a Guava easyCyte HT (Merck/Millipore, Darmstadt, Germany) using Guava InCyte software, respectively. Red blood cell green fluorescence (525 nm) was recorded using a GFP-positive gate that was set to exclude 99% of WT erythrocytes, as described previously [24].

#### Mapping of the *MommeD41* mutation and linkage analysis

The Line3C [39] was used for mapping and linkage analysis. *MommeD41* heterozygous mice were backcrossed to Line3C twice and phenotyped using flow cytometry. DNA collected from tail tissue of WT (*n* = 12) and *MommeD41* heterozygotes (*n* = 11) was used to perform linkage analysis using the Illumina GoldenGate genotyping assay exactly as described previously [25, 39]. Only samples with a call rate > 95 were accepted, and a linked interval was identified based on LOD score. A LOD score of > 5 was found for chromosome 16 (Additional file 1: Table S1).

#### Whole exome deep sequencing

Genomic DNA isolated from tail of one WT and one MommeD41 heterozygote were used for exome capture using the RocheNimbleGen reagents (SeqCap EZ Mouse exome, version beta 2, 110603_MM9_exome_rebal_2EZ_HX1, Madison, WI, USA) according to the Illumina optimized RocheNimbleGen SeqCap User’s guide. Libraries were sequencing using an Illumina GAIIx platform and reads were aligned to the mouse reference genome (build 37, mm9) as described previously [25]. Varscan output was used to identify likely heterozygous mutations and the *Morc3*^*MD41*^ mutation was validated in additional heterozygous mice using Sanger sequencing.

#### Mouse embryonic stem cell (mESC) derivation and culture

mESCs were generated from WT and *Morc3*^*MD41/MD41*^ preimplantation embryos and were cultured on 0.1% gelatin without feeders in mESC medium [Knockout DMEM (10829-018; Gibco), 10% FBS (DE14-801F; BioWhittaker), NEAA (11140; Gibco), l-Glutamine (25030-123; Gibco), Sodium Pyruvate (11360; Gibco), 2-Mercaptoethanol (31350; Gibco) and Leukemia Inhibitory Factor (ESG1107; Millipore)] plus MEK inhibitor PD0325901 (1 mM) and GSK3 inhibitor CHIR99021 (3 mM, Axon Medchem). Cell cultures tested negative for mycoplasma on a regular basis.

#### Genotyping

Cell or tissues were incubated overnight in DNA lysis buffer (50 mM TrisHCl pH 8.0, 5 mM EDTA, 2%SDS) supplemented with Proteinase K (Invitrogen AM2548) at 55 ℃. After RNA digestion with RNAseA (Thermo scientific EN0531) 30 min at 37 ℃, genomic DNA was precipitated using 5 M NaCl, 1 volume isopropanol and 70% Ethanol. DNA was eluted in water and used subsequently as template for genotyping using DreamTaq Polymerase (Thermo scientific EP0705) and the following primers: TGTCCAGCCCTGTATGTTGG (forward) and ACATAGTGAATCCCAGCAGAGC (reverse). PCR products were then sequenced with Sanger sequencing.

#### RNA isolation and RT-qPCR analysis

Total RNA was isolated with QIAzol (5346994; Qiagen). About 1 mg of total RNA was used for reverse transcription using RevertAid H Minus First Strand cDNA Synthesis Kit (K1632; Thermo). RT-qPCR was performed in triplicate on a C1000TM Thermal cycler (Bio-Rad) with SYBR Green (170-8887; Bio-Rad). Expression data was normalized to b-actin. Primer sequences are provided in Additional file 6: Table S5.

#### Alkaline phosphatase staining

mESCs were controlled for pluripotency using the StemAb Alkaline phosphatase staining Kit II (00-0055; Stemgent). Briefly mESc cells cultured on 0.1% gelatin were washed with PBS twice and fixed for 5 min with 0.5 mL fixative solution at room temperature. Fixative solution was rinsed with PBS before incubation of the cells with staining solution for 15 min at room temperature. Staining solution was then rinsed with PBS and cells were observed for purple coloration under binocular.

#### Western blot

Cells were lysed in Cell Lysis buffer (20 mM triethanolamine (T1377; Sigma), 0.14 M NaCl, 0.1% Sodium deoxycholate (D6750; Sigma), 0.1% SDS, 0.1% Triton X-100) with Protease Inhibitor Cocktail (27368400; Roche), Phosphatase Inhibitor Cocktail (04906837001; Roche) and 10 mM *N*-Ethylmaleimide (NEM) on ice. BCA kit (23225; Thermo) was used to measure protein concentration. Equal amounts of total cell extracts were loaded on a NuPAGE gel (4–12%, NP0321; Thermo), and transferred to a Nitrocellulose Blotting Membrane (10600016; Life Sciences). The following primary antibodies were used: Morc3 (Rockland; 100-401-N96S 1:1000) and Tubulin (T6199; Sigma, 1:5000). Donkey anti-Rabbit 800CW (926-32213; Li-Cor, 1:5000), Goat anti-Rabbit 800CW (926-32211; Westburg, 1:5000), Donkey anti-mouse 680RD (926-68072; Li-Cor, 1:5000) were used as secondary antibodies. Membranes were analyzed on Odyssey (Westburg).

#### RNA-seq

Total RNA was isolated as described above and standard RNA-seq was performed at BGI. Sample preparation after rRNA depletion was performed using NEB Next Ultra Directional RNA Library Prep Kit for Illumina (E7420S/L; NEB) according to the protocol. Libraries were sequenced with 100 bp pair-end (PE) reads on a HiSeq 2500.

#### Morc3 ChIP-qPCR

Cells were cross linked with 1% formaldehyde M134-200ML; VWR) for 8 min at room temperature and glycine (125 mM; G8790-1 KG; Sigma) was used to quench cross-linking for 5 min. Cells were washed twice with cold PBS and lysed in ChIP Buffer 1 (10 mM Tris HCl (pH 8.0), 0,25% Triton X-100, 10 mM EDTA, 0.5 mM EGTA, Protease Inhibitor Cocktail (05056489,001; Roche) and 1 mM PMSF) for 15 min on a rotator at 4 °C. After centrifugation 5 min at 1400*g* at 4 °C, cells were resuspended in ChIP Buffer 2 (10 mM Tris–HCl (pH 8.0), 200 mM NaCl, 10 mM EDTA, 0.5 mM EGTA, Protease Inhibitor Cocktail (05056489001; Roche) and 1 mM PMSF). After centrifugation 5 min at 1400*g* at 4 °C, cells were resuspended in ChIP Buffer 3 (10 mM Tris–HCl (pH 8.0), 10 mM EDTA, 0.5 mM EGTA, 0,1% SDS, Protease Inhibitor Cocktail (05056489001; Roche) and 1 mM PMSF and sonicated with Covaris S2 (Duty cycle: 10%, Intensity:5, Cycle/Burst:200 to obtain fragment between 200 and 600 bp. Sheared chromatin was centrifuged at 12,000*g* for 10 min at 4 °C to discard the pellets. The supernatant was then diluted 10 times with ChIP dilution buffer (16.7 mM Tris–HCl (pH8), 0.01% SDS, 1.1% TritonX-100, 1.2 mM EDTA, 167 mM NaCl) and incubated overnight with 5 µg Morc3 antibody (Rockland; 100-401-N96S) or Rabbit IgG (Abcam ab37415). An input fraction for each sample was retained for downstream analysis. Samples were then incubated with Dynabeads Protein A (Invitrogen 10001D) for at least 4 h rotating at 4 °C. After immunoprecipitation beads were washed with low-salt washing buffer (0.1% SDS, 1% Triton X-100, 2 mM EDTA, 20 mM Tris–HCl (pH 8.1), 150 mM NaCl), high-salt washing buffer (0.1% SDS, 1% Triton X-100, 2 mM EDTA, 20 mM Tris–HCl (pH 8.1), 500 mM NaCl), LiCl washing buffer (0.25 M LiCl, 1% NP40, 1% deoxycholate, 1 mM EDTA, 10 mM Tris–HCl (pH 8.1)) and TE buffer (10 mM Tris–HCl (pH 8.0), 1 mM EDTA). IP DNA and Input DNA samples were extracted with phenol–chloroform–isoamyl alcohol (15593049; Fisher Scientific). Quantitative PCR was performed using the primer sequences provided in Additional file 6: Table S5.

#### Trim28 ChIP-qPCR

Trim28 ChIP was performed as described above. Chromatin was immunoprecipitated with 5µL of Trim28 antibody (Abcam ab22553). DNA was subsequently used for quantitative PCR using the primer sequences provided in Additional file 6: Table S5.

#### Quantitative H3K9me3 ChIP-seq

Cells were cross linked with 1% formaldehyde (M134-200ML; VWR) for 8 min at room temperature and glycine (125 mM; G8790-1 KG; Sigma) was used to quench cross-linking for 5 min. Cells were washed twice with cold PBS and lysed in NP Buffer (150 mM NaCl, 50 mM Tris–HCl (pH 7.5), 5 mM EDTA, 0.5% NP-40, 1% Triton X-100, Protease Inhibitor Cocktail (05056489001; Roche)). Nuclei were sheared by sonication (Covaris). For each H3K9me3 ChIP-seq experiment, 25 µg of sample chromatin was mixed with 50 ng spike-in *Drosophila* chromatin (53,083; Active Motif). Mixture of experimental chromatin and spike-in chromatin was then incubated with a mix containing 4 µg of H3K9me3 antibody (abcam ab8898) and 2 µg of spike-in antibody (104,597; Active Motif) at 4 °C overnight. The next day, Protein A Sepharose beads (175280-01; GE Health Care) were first blocked with 1 mg/mL BSA (10484; Affymetrix) and then added to each chromatin-antibody mix and incubated at 4 °C for at least 3 h. After immunoprecipitation, beads were washed with low-salt washing buffer (0.1% SDS, 1% Triton X-100, 2 mM EDTA, 20 mM Tris–HCl (pH 8.1), 150 mM NaCl), high-salt washing buffer (0.1% SDS, 1% Triton X-100, 2 mM EDTA, 20 mM Tris–HCl (pH 8.1), 500 mM NaCl), LiCl washing buffer (0.25 M LiCl, 1% NP40, 1% deoxycholate, 1 mM EDTA, 10 mM Tris–HCl (pH 8.1)) and TE buffer (10 mM Tris–HCl (pH 8.0), 1 mM EDTA). DNA was extracted using phenol–chloroform–isoamylol (15,593–049; Life Technologies). Samples were sequenced at Macrogen on HiseqX with 150 bp paired-end (PE) reads.

#### Assay for transposase accessible chromatin

ATAC-seq libraries were prepared as previously described [40]. In brief, 50,000 mouse embryonic stem cells were resuspended in 50 µL transposition mix (Nextera) and incubated for 30 min at 37 °C. Libraries were amplified by PCR with barcoded Nextera primers and sequenced on BGISEQ-500 with 150 bp pair-end (PE) reads.

### CRISPR line

#### V6.5 mESC culture

WT V6.5 mESCs and Morc3 null mutants derived from V6.5 mESCs were cultured on mouse embryonic fibroblasts (feeder cells). Cell culture media composed of KnockOut DMEM (10829-018, Invitrogen), 15% Hyclone fetal bovine serum (SH3007003, GE), 1X Penicillin–Streptomycin–Glutamine (Gibco, 10378016), 50 µg/mL primocin (ant-pm-2, Invivogen), 1X MEM non-essential amino acids (Gibco, 11140050), 1000 U/mL ESGRO mouse LIF (Millipore, ESG1106) and 55 µM beta-Mercaptoethanol (21985-023, Invitrogen) was used. Before RNAseq or ChIPseq, cells were transitioned off feeders and onto gelatin for two passages.

#### CRISPR/Cas9 genome editing

To generate a Morc3 mutant line, a guide RNA (gRNA) targeting exon 2 of *Morc3* gene was designed and cloned into PX330 vector (http://www.addgene.org/42230/) (https://pubmed.ncbi.nlm.nih.gov/23287718/). 50,000 cells were plated in one well of a 6-well plate 24 h prior to transfection. ~ 3 µg of gRNA plasmid and ~ 1 µg of pMax-GFP were cotransfected using Lipofectamine 2000. Two days after transfection, GFP + cells were sorted into a 96-well plate and allowed to grow into colonies over several days. Candidate lines were screened with the Surveyor Assay. To determine the precise mutations, genomic DNA was extracted from about 1 million cells using Quick-DNA Microprep Kit (ZYMO RESEARCH, D3021). 1µL of the genomic DNA was used as PCR template for genotyping using Phusion High-Fidelity DNA Polymerase (NEW ENGLAND BioLabs, M0530L). gRNAs and genotyping primers are listed in Additional file 6: Table S5.

#### Western blot

Cells were lysed in NuPAGE LDS Sample Buffer with NuPAGE reducing agent and were heated at 70 °C for 10–20 min. Equal amounts of cell extracts from WT and mutants were loaded on a 4–12% NuPAGE Bis–Tris gel. The gel was ran in 1X MOPS SDS running buffer at 200 V for 50–60 min. It was then transferred to a PVDF membrane at 30 V for 60 min in 1X NuPAGE transfer buffer. NuPAGE antioxidant was added to the running and transfer buffer. An ice block was placed in the gel box to keep the transfer buffer cool. The membrane was cut accordingly and blocked with 10% goat serum for 1 h at room temperature with gentle agitation. Then, the membrane was incubated in 1:3000 dilution of Morc3 antibody in 10% goat serum for 1 h at room temp with gentle agitation. After primary antibody incubation, the membranes were washed with 1xTBST for 5 min. Total 4 washes were performed and then the membrane was incubated in 1:3000 dilution of goat–anti-rabbit antibody in 10% goat serum for 1 h at room temp with gentle agitation. The membrane was then washed 4 times with 1xTBST for 5 min each, stained with ECL (1:1 solutions A and B) for 2 min, and imaged with the "camera station" instead of film.

#### RNA-seq

RNA from three independently passaged *Morc3*^*–/–*^ lines was extracted using RNeasy Mini Kit (74104, Qiagen) and libraries were prepared using TruSeq Stranded mRNA Library Prep kit (20020595, Illumina). Libraries were then sequenced with 100 bp pair-end (PE) reads on a NovaSeq 6000.

#### MORC3 and H3K9me3 ChIPseq

V6.5 mouse embryonic stem cells (mESCs) were cultured on mouse embryonic fibroblasts (MEFs) with fetal bovine serum (FBS) and leukemia inhibitory factor. To fix cells for ChIP, mESCs were grown for up to two passages on gelatin and without MEFs, then grown to ~ 80% confluency. Cells were harvested with 0.25% Tryspin, quenched with FBS containing media, washed with PBS and counted. Fixation was performed with 1.5% formaldehyde in PBS for 14 min with nutation then quenched with 120 mM glycine for 5 min. Fixed cells were pelleted and washed with PBS twice, counted and 8 million cell aliquots were flash frozen. Eight million cells per replicate were thawed on ice and resuspended in 1 mL 10 mM Tris pH 8.0, 0.25% Triton X-100, 10 mM EDTA, 0.5 mM EGTA, 1 mM PMSF, and Roche Complete EDTA-free Mini Protease inhibitor cocktail and then incubated with rotation for 15 min at room temperature. Nuclei were pelleted by centrifugation at 1500×*g* for 5 min at 4 °C. The nuclei were resuspended in 10 mM Tris pH 8.0, 200 mM NaCl, 10 mM EDTA, 0.5 mM EGTA, 1 mM PMSF and protease inhibitor cocktail, incubated for 10 min at room temperature with rotation, and then centrifuged again. Nuclei were then resuspended in 10 mM Tris pH 8.0, 10 mM EDTA, 0.5 mM EGTA, 0.1% SDS, 1 mM PMSF and protease inhibitor cocktail, and disrupted by sonication at high intensity. Two replicates for MORC3 ChIP were sonicated using Bioruptor with the following parameters: 30 s on/30 s off for 20 min (10 min actual sonication and 10 min dormant). Ice was replaced every 5 min. Another two replicates for MORC3 ChIP and all replicates for H3K9me3 ChIP were sonicated using the following parameters: time: 430 s, Duty cycle: 10%, Intensity: 5, Cycle/Burst: 200. For analysis, peaks common between all replicates were used. Sonicated lysate was cleared by centrifugation at 16,000×*g* for 10 min at 4 °C, and the supernatant was used for ChIP. Samples were diluted with an equal volume of 10 mM Tris pH 8.0, 10 mM EDTA, 0.5 mM EGTA, 0.1% SDS, 1 mM PMSF and protease inhibitor cocktail. The samples were then precleared with 30 μL of protein A magnetic Dynabeads (Thermo Fisher Scientific), which had been washed with 16.7 mM Tris pH 8.0, 0.01% SDS, 1.1% Triton X-100, 1.2 mM EDTA, and 167 mM NaCl before use, followed by incubation for 2 h at 4 °C. The beads were then collected on a magnet, and the supernatant was retained. Once 10% of the sample was saved for input, the remaining sample was treated with 5 μg of requisite target antibody (MORC3: anti-MORC3 antibody generated in collaboration with Rockland Immunochemicals; H3K9me3 abcam 8898). Samples were incubated overnight at 4 °C with rotation. The next day, 100 μL of protein A beads, which had been washed with 16.7 mM Tris pH 8.0, 0.01% SDS, 1.1% Triton X-100, 1.2 mM EDTA, and 167 mM NaCl before use, were added to each sample, followed by incubation for another 2 h. The beads were washed twice for 4 min each time with rotation with 50 mM Hepes pH 7.9, 1% Triton X-100, 0.1% deoxycholate, 1 mM EDTA, and 140 mM NaCl; washed twice for 4 min each time under rotation with 50 mM Hepes pH 7.9, 0.1% SDS, 1% Triton X-100, 0.1% deoxycholate, 1 mM EDTA, and 500 mM NaCl; and then washed twice for 4 min each time under rotation with 500 μL of 10 mM Tris pH 8.0 and 1 mM EDTA. The purified DNA was eluted by incubation with elution buffer (100 μL of 50 mM Tris pH 8.0, 1 mM EDTA, and 1% SDS) at 65 °C for 10 min. Eluent was collected on a magnetic rack, and the beads were resuspended with 150 μL of elution buffer and then incubated at 65 °C for 10 min. The two eluents were pooled and de-cross-linked by incubation at 65 °C overnight, as were the inputs from day 1 after being thawed. The samples were brought to room temperature and then warmed to 37 °C and incubated with 10 μg RNase A (Qiagen). The samples were then treated with 15 μg of proteinase K and incubated for 2 h at 56 °C. Finally, after cooling, the samples were purified with Qiagen MinElute columns. Purified DNA was quantified with Qubit High-Sensitivity reagent (Thermo Fisher Scientific), and libraries were generated with the Ovation Ultralow Library System Kit (Nugen) using 10 ng of input DNA. MORC3 ChIP-seq libraries were sequenced with 50 bp single-end reads and H3K9me3 ChIP-seq libraries were sequenced with 100 bp single-end reads.

## Quantification and statistical analysis

### RNA-seq analysis

Quality assessment of the raw sequencing reads was done using FastQC v0.11.8 (http://www.bioinformatics.babraham.ac.uk/projects/fastqc). Adapters were removed by TrimGalore v0.4.5 (https://www.bioinformatics.babraham.ac.uk/projects/trim_galore/) using a default parameters and stringency of 3 for paired-end Illumina reads, after which, quality filtering was performed by the same software. Reads smaller than 20bps and with quality score less than 25 were discarded, after which a final quality assessment of the filtered reads was done with FastQC to identify possible biases left after filtering. For gene expression analysis, the remaining reads were mapped to the mouse reference genome (GRCm38/mm10) using the STAR aligner v2.7.0e [41] using the following parameters for unique mapping of reads: STAR—alignEndsType EndToEnd—genomeDir [$genome_location]—readFilesIn [$input files]—outFilterType BySJout—outFilterMultimapNmax 1 –outFilterMismatchNmax 999—outFilterMismatchNoverReadLmax 0.05—alignIntronMin 20—alignIntronMax 1000000—alignMatesGapMax 1000000—outFileNamePrefix [$outprefix]—outSAMtype BAM SortedByCoordinate—outSAMunmapped Within—outReadsUnmapped Fastx—outFilterIntronMotifs RemoveNoncanonical. Before mRNA quantification, duplicated reads were marked with Picard tools v2.17 (http://broadinstitute.github.io/picard/). Quantification was done by HTSeq-count v0.91 [42], using the ENSEMBL annotation (downloaded from: ftp://ftp.ensembl.org/pub/release-99/gtf/mus_musculus/Mus_musculus.GRCm38.99.gtf.gz) with the option “–stranded reverse”. Statistical analysis was done using DESeq2 v1.2.0 [43] (R package). Volcanoplot were generated using VolcanoseR [44]. For transposable elements analysis, trimmed reads were mapped to the mouse reference genome (GRCm38/mm10) using the STAR aligner v2.7.0e with the same parameters as above for unique mapping. Annotation for TEs was obtained from: http://labshare.cshl.edu/shares/mhammelllab/www-data/TEtranscripts/TE_GTF/GRCm38_Ensembl_rmsk_TE.gtf.gz. TEs were also analyzed by allowing trimmed reads to multimap and then using TEtranscripts [27] to quantify expression changes between mutants and WT. For multimapping, the following STAR command was used: STAR—alignEndsType EndToEnd—genomeDir [$genome_location]—readFilesIn [$input files]—readFilesCommand zcat—outFilterType BySJout—outFilterMultimapNmax 5000—outFilterMismatchNmax 999—outFilterMismatchNoverReadLmax 0.05—alignIntronMin 20—alignIntronMax 1000000—alignMatesGapMax 1000000—outFileNamePrefix [$outprefix]—outSAMtype BAM SortedByCoordinate—outSAMunmapped Within—outReadsUnmapped Fastx—outFilterIntronMotifs RemoveNoncanonical—seedSearchStartLmax 30—alignTranscriptsPerReadNmax 30000—alignWindowsPerReadNmax 30000—alignTranscriptsPerWindowNmax 300—seedPerReadNmax 3000—seedPerWindowNmax 300—seedNoneLociPerWindow 1000. Quantification was done using TEtranscripts in the multimap mode. Heatmaps and profiles were generated using Deeptools [45].

### ATAC and ChIP-seq analysis

Quality assessment of the raw sequencing reads was done using FastQC v0.11.6 (http://www.bioinformatics.babraham.ac.uk/projects/fastqc). Adapters were removed by TrimGalore v0.4.5 using default parameters for single and paired-end reads. Trimmed reads were aligned to the mouse reference genome (build mm10) using bowtie 2 v2.3.4 [46] with the parameter “—very-sensitive-local” for ChIP-seq and the parameter “—very-sensitive” for ATAC-seq. Peaks were called using MACS2 v2.1.1 [47] with parameters “-g mm” and “qvalue 0.05” for Morc3 ChIP-seq and ATAC-seq and SICER 2 (1.0.3) [48] identify H3K9me3 enriched domains. For ChIP-seq peaks were called using input fractions as control. For downstream analysis, only peaks overlapping between replicates were used. Peaks were then annotated with ChIPseeker [49] and HOMER [50]. Differential ATAC peaks were determined with the Homer function getDifferentialPeaks. To generate the tracks and the heatmap and profile plots, deepTools version 3.1.3 [45] was used. For the plot profiles, a bin size of 10 bps and a smoothing window of 40 bps was chosen. H3K9me3 coverage analysis over transposable elements was performed with bedtools intersect (v2.28) [51] using the UCSC RepeatMasker track (mm10). Reads overlapping with transposable elements were normalized for the library size and by the coverage in the input sample and then sorted and grouped by repetitive element names. H3K9me3 enrichment over input were plotted using the R packages pheatmap v1.0.12 and ggplot2 v3.1.1. Diffbind (3.0.15) was used to measure H3K9me3 differential peaks [52, 53].

## Supplementary Information

**Additional file 1: Figures S1–S12.** List of supplementary figures, titles and legends.

**Additional file 2: Table S1.** Illumina GoldenGate SNP genotyping assay.

**Additional file 3: Table S2.** Whole exome sequencing result.

**Additional file 4: Table S3.** Annotation of MORC3 ChIP-seq peaks shared across replicates.

**Additional file 5: Table S4.** Annotation of H3K9me3 ChIP-seq peaks shared across replicates.

**Additional file 6: Table S5.** List of primers.

## Data Availability

The data used in this study have been deposited in the NCBI Gene Expression Omnibus (GEO) under accession code GSE173917. TRIM28 ChIP-seq in Additional file 1: Figure S5 was obtained from GSM1555121. H3K4me3 ChIP-seq in Additional file 1: Figure S6 was obtained from GSM769008.

## Abbreviations

MORCs: Microrchidia proteins
*MommeD*: *Modifiers of murine metastable epialleles Dominant*
TEs: Transposable elements
mESCs: Mouse embryonic stem cells
ERVs: Endogenous retroviruses
5mC: 5-Methylcytosine
HUSH: Human silencing hub
IAPs: Intracisternal A particles
WT: Wild type
*Su(var)*: Suppressor of variegation
*E(var*): Enhancer of variegation
TSS: Transcription start sites
DAL: Differentially accessible loci
