# Supplementary material for: The role of MORC3 in silencing transposable elements in mouse embryonic stem cells
Source: Epigenetics Chromatin. 2021 Oct 27;14:49. doi: 10.1186/s13072-021-00420-9 (PMC8555065; doi:10.1186/s13072-021-00420-9)
Supplement: Supplementary file 1 — Additional file 1: Figures S1–S12. List of supplementary figures, titles and legends. [file 13072_2021_420_MOESM1_ESM.pdf]

Additional file 1: Figures S1-S12

Figure S1:

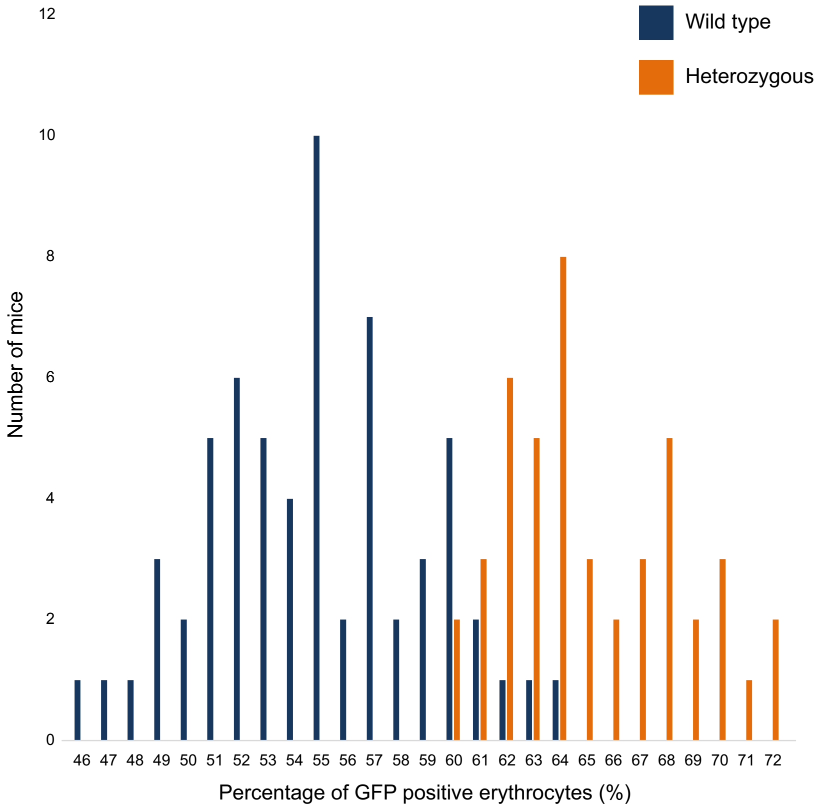

Figure S1: Distribution of GFP expression in the *MommeD41* colony. The percentage of red blood cells expressing GFP was assessed in 107 mice from the *MommeD41* colony at weaning, and grouped by genotype for the *Morc3* mutation. Wild types are shown in blue and heterozygotes are shown in orange.

Figure S2:

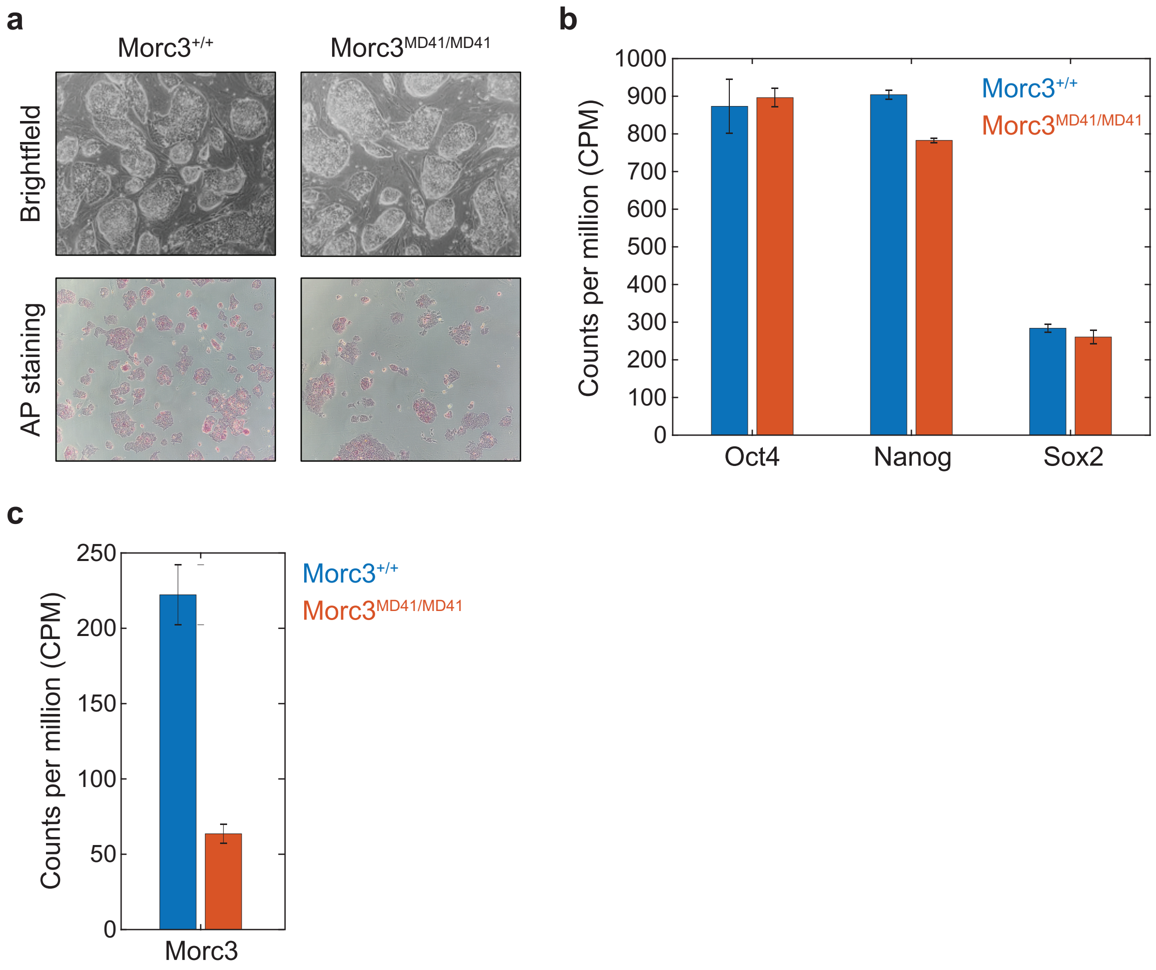

Figure S2: Characterization of *Morc3*$^{MD41/MD41}$ mESCs (MommeD line). **a** Representative images of cellular morphology and alkaline phosphatase (AP) staining of the WT and the *Morc3*$^{MD41/MD41}$ derived mESCs grown in 2i and serum. **b** Pluripotent gene expression levels in WT and the *Morc3*$^{MD41/MD41}$. Error bars represent 1 standard deviation. **c** RNA-seq validates the downregulation of *Morc3* in *Morc3*$^{MD41/MD41}$. Error bars represent 1 standard deviation.

Figure S3:

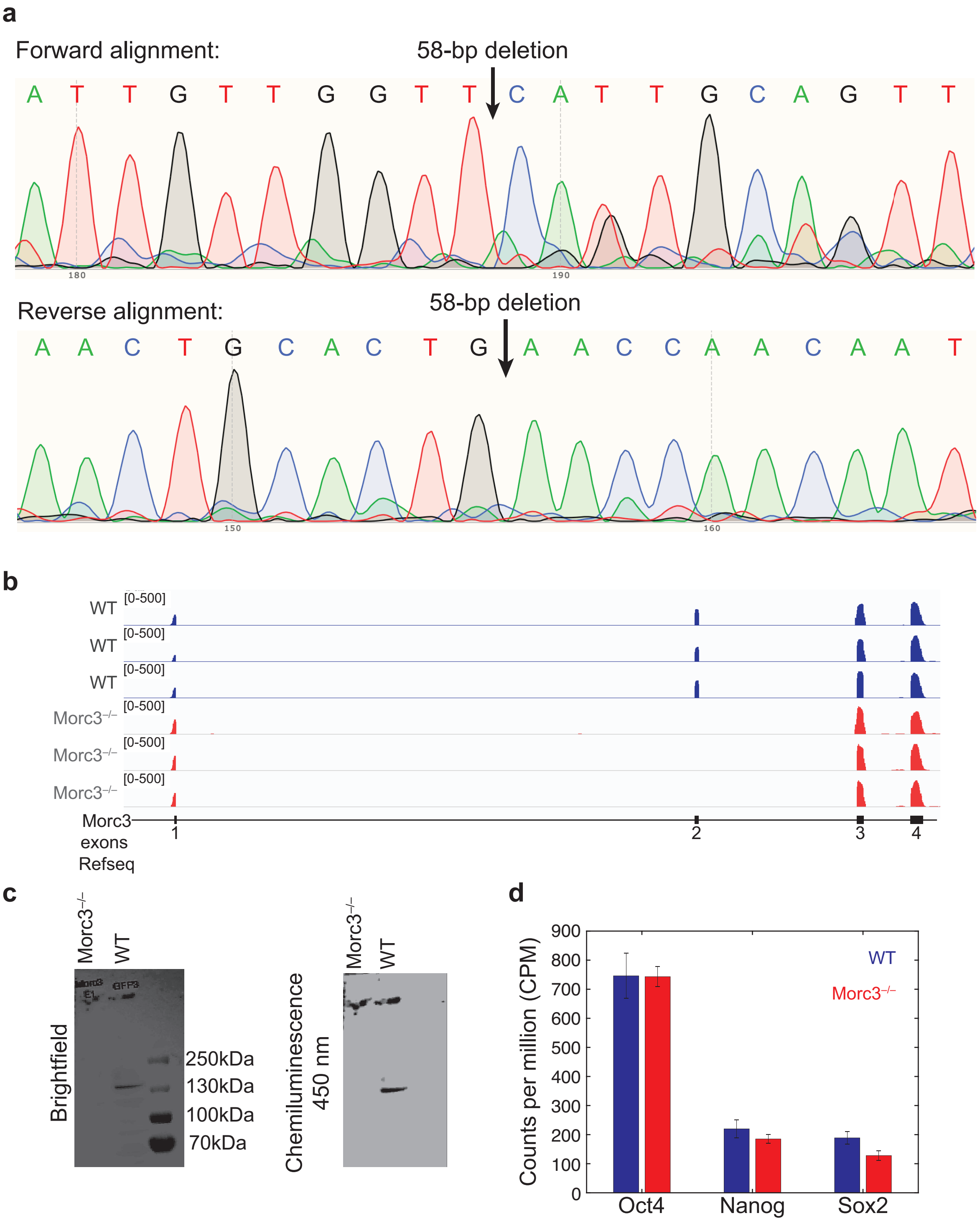

Figure S3: Characterization of *Morc3*$^{-/-}$ mESCs (CRISPR line). **a** Sanger sequencing shows a 58-bp deletion in exon 2 of *Morc3* gene. **b** Genome browser shot of mapped RNAseq reads shows absence of exon 2 in the *Morc3*$^{-/-}$ mutant lines. **c** Western blot in *Morc3*$^{-/-}$ and WT mESC lines shows absence of MORC3 protein in the mutant line. **d** Pluripotent gene expression levels in WT and the *Morc3*$^{-/-}$. Error bars represent 1 standard deviation.

Figure S4:

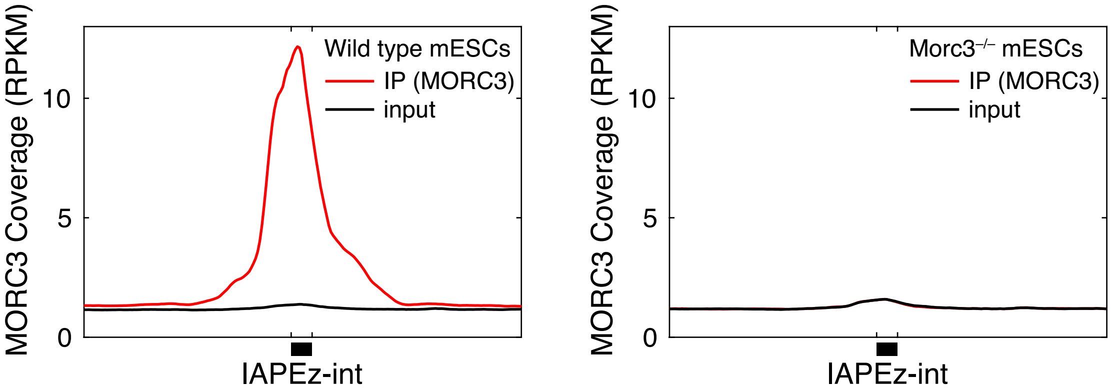

Figure S4: MORC3 enrichment at IAPEz-ints in WT mESCs (left) and *Morc3*$^{-/-}$ mESCs (right).

Figure S5

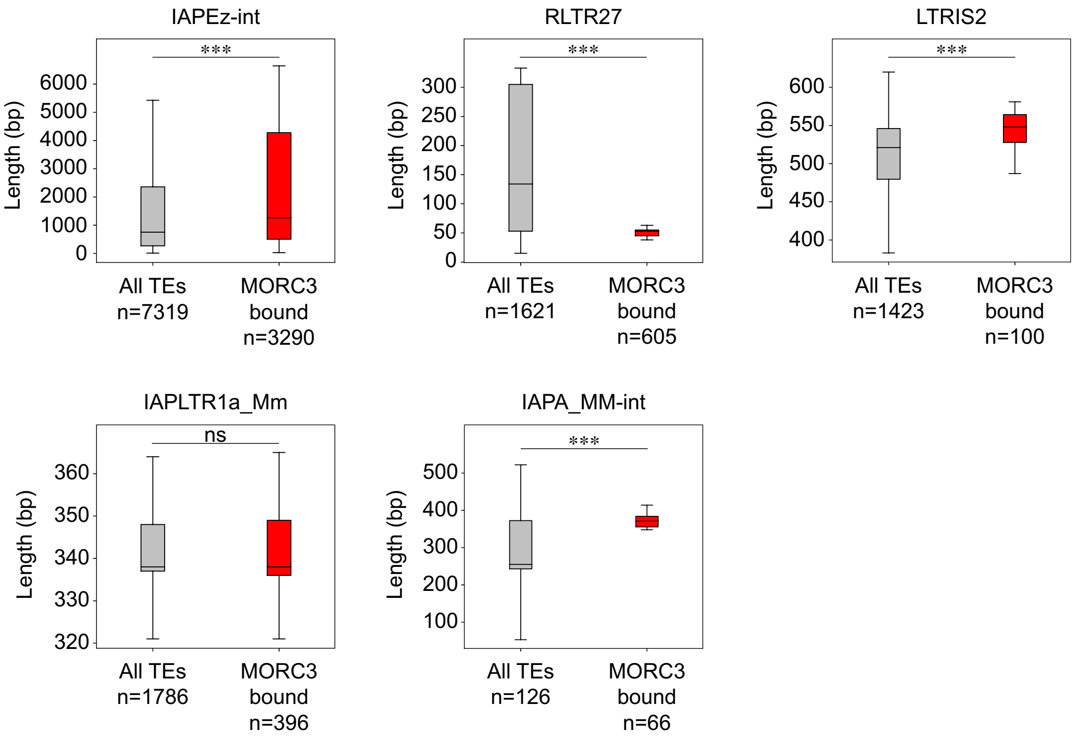

Figure S5: Boxplots comparing the length of TEs bound by MORC3 with the length of all TEs in that subfamily. Mann–Whitney U test was used to test for significance (p-value = 9.33e-48 for IAPEz-int, p-value = 3.2e-68 for RLTR27, p-value = 2e-11 for LTRIS2, p-value = 0.5 for IAPLTR1a_Mm and p-value = 1.4e-05 for IAPA_MM-int)

Figure S6

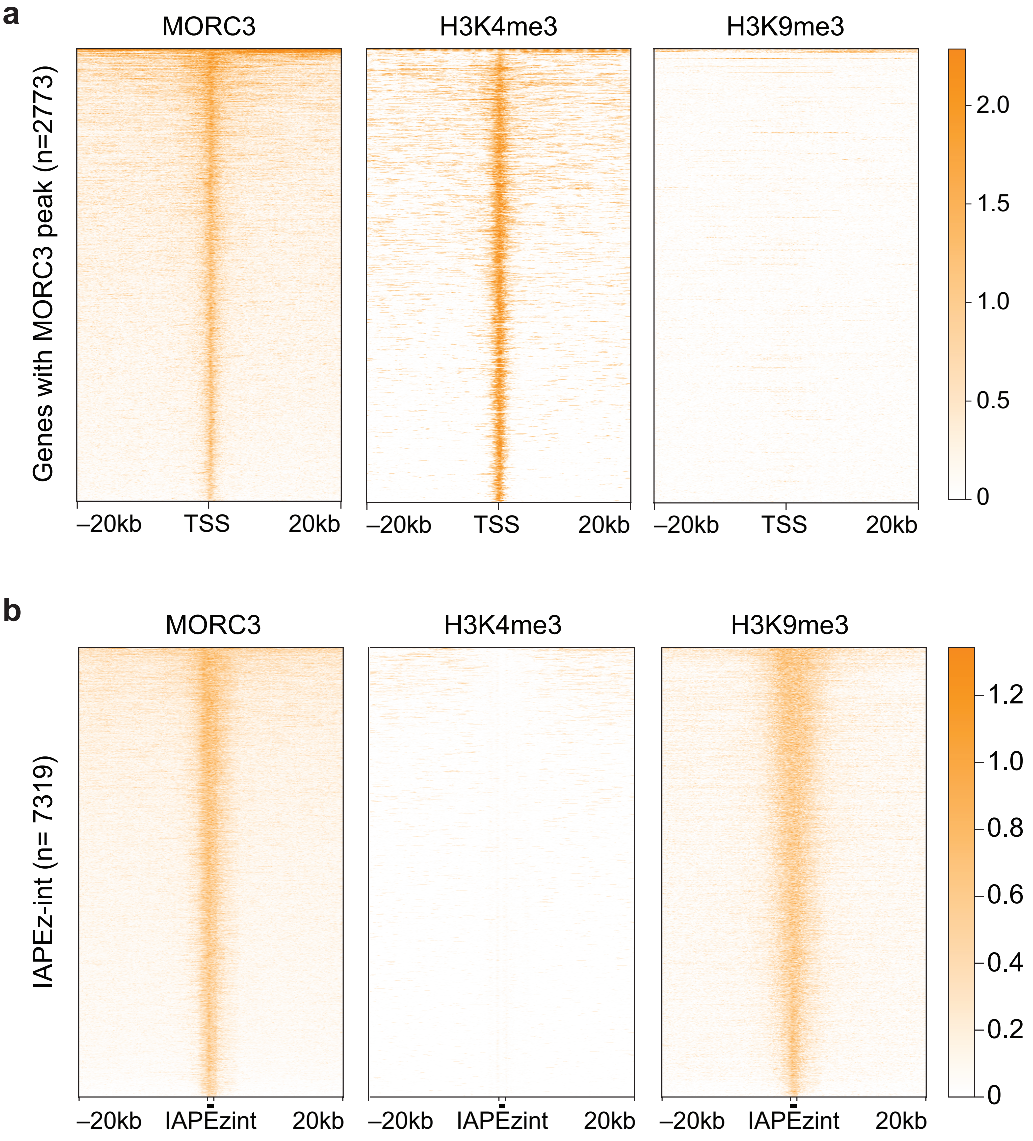

Figure S6: Coverage of MORC3, H3K4me3 and H3K9me3 at MORC3 bound promoters (**a**) and at IAPEz-int (**b**).

Figure S7

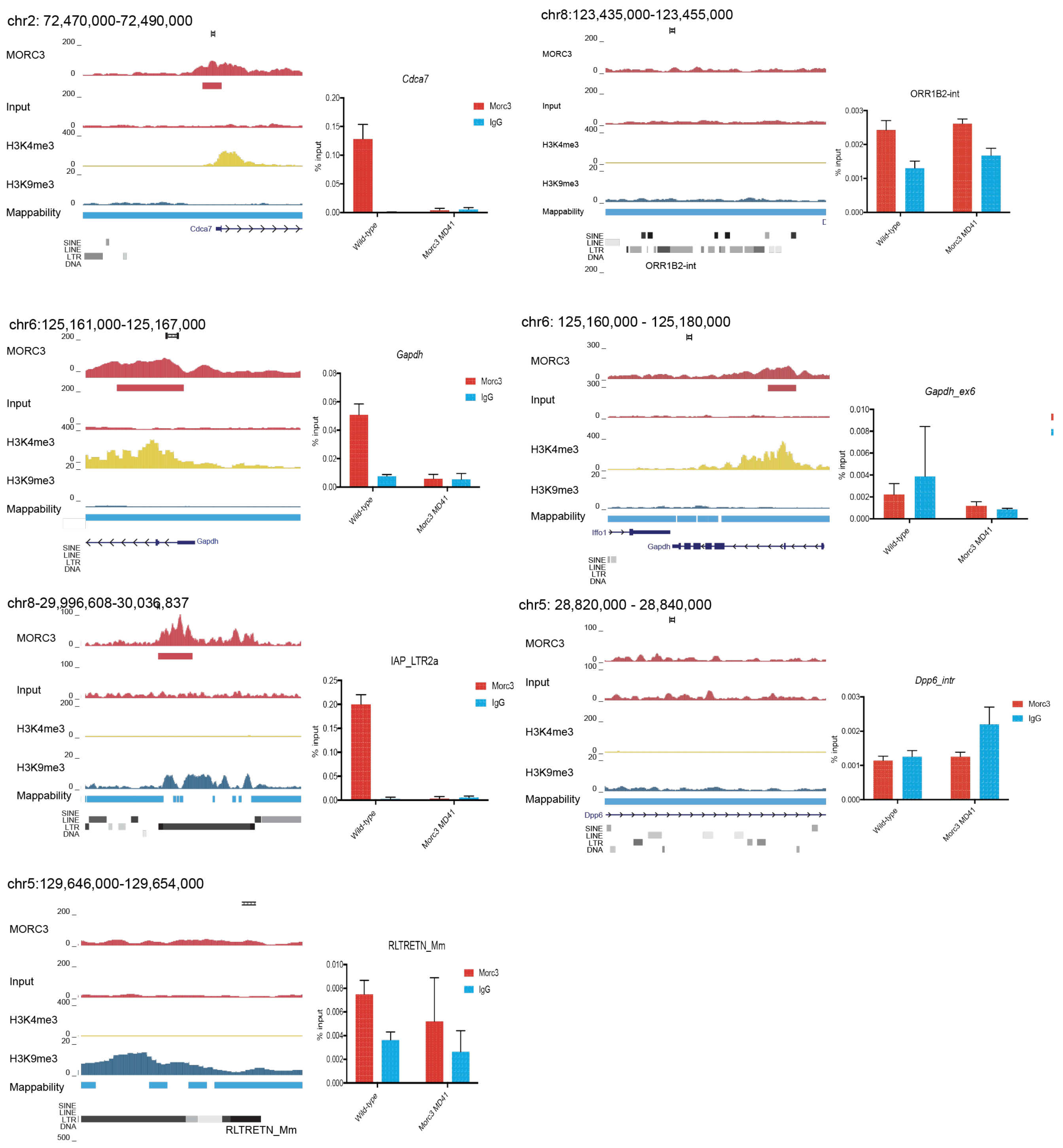

Figure S7: MORC3 ChIP-qPCR to validate the presence of MORC3 at the *Cdca7* promoter, at exon 1 of *Gapdh* and at an IAPLTR2a. RLTRETN_Mm, ORR1B2-int, *Gapdh* exon6 and *Dpp6* intron are loci where MORC3 is absent and were used as negative control for MORC3 ChIP-qPCR.

Figure S8:

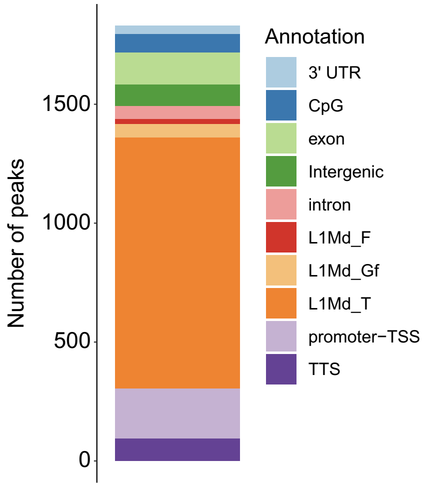

Figure S8: Distribution of MORC2A ChIP-seq peaks over genomic features.

Figure S9:

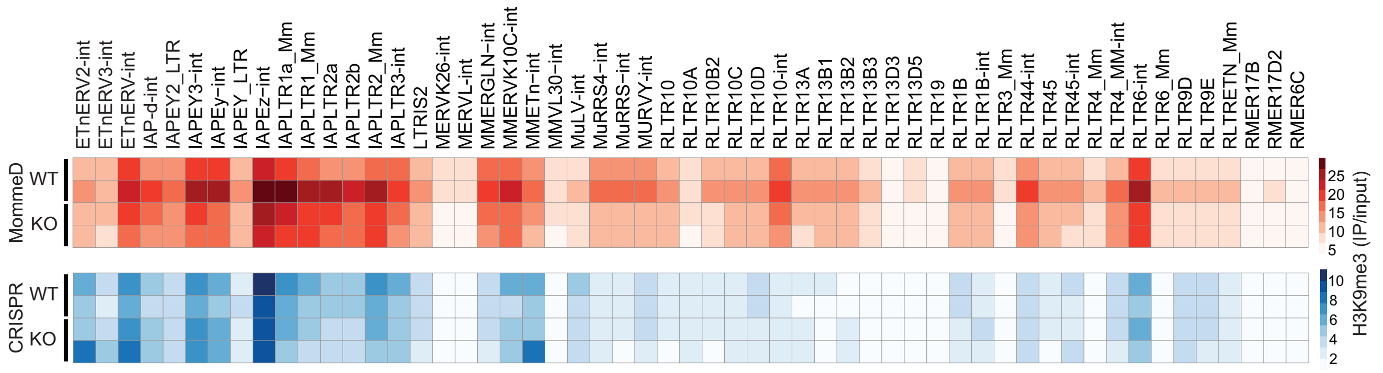

Figure S9: Heatmaps showing the enrichment of H3K9me3 over TEs in MommeD and CRISPR lines.

Figure S10:

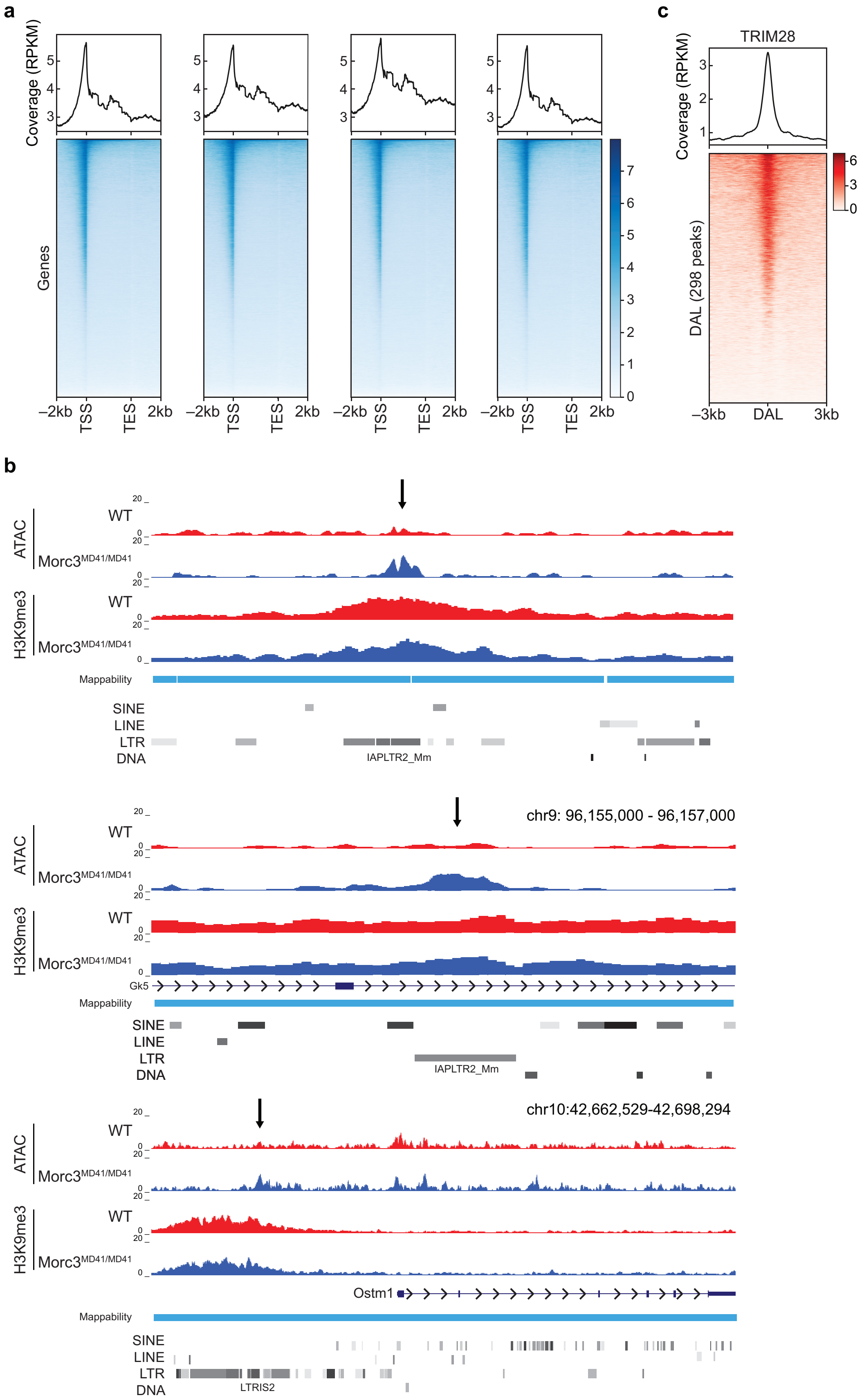

Figure S10: a Meta plot and heatmaps showing enrichment of ATAC-seq read coverage around TSS of mouse genes. Coverage is measured in rpkm. **b** Metaplot and heatmaps showing the enrichment of TRIM28 at DAL. **c**

Representative genome browser tracks showing examples of DAL. A black arrow indicates a locus identified as differentially accessible. DAL are covered with H3K9me3 as indicated by the H3K9me3 ChIP-seq tracks.

FigureS11:

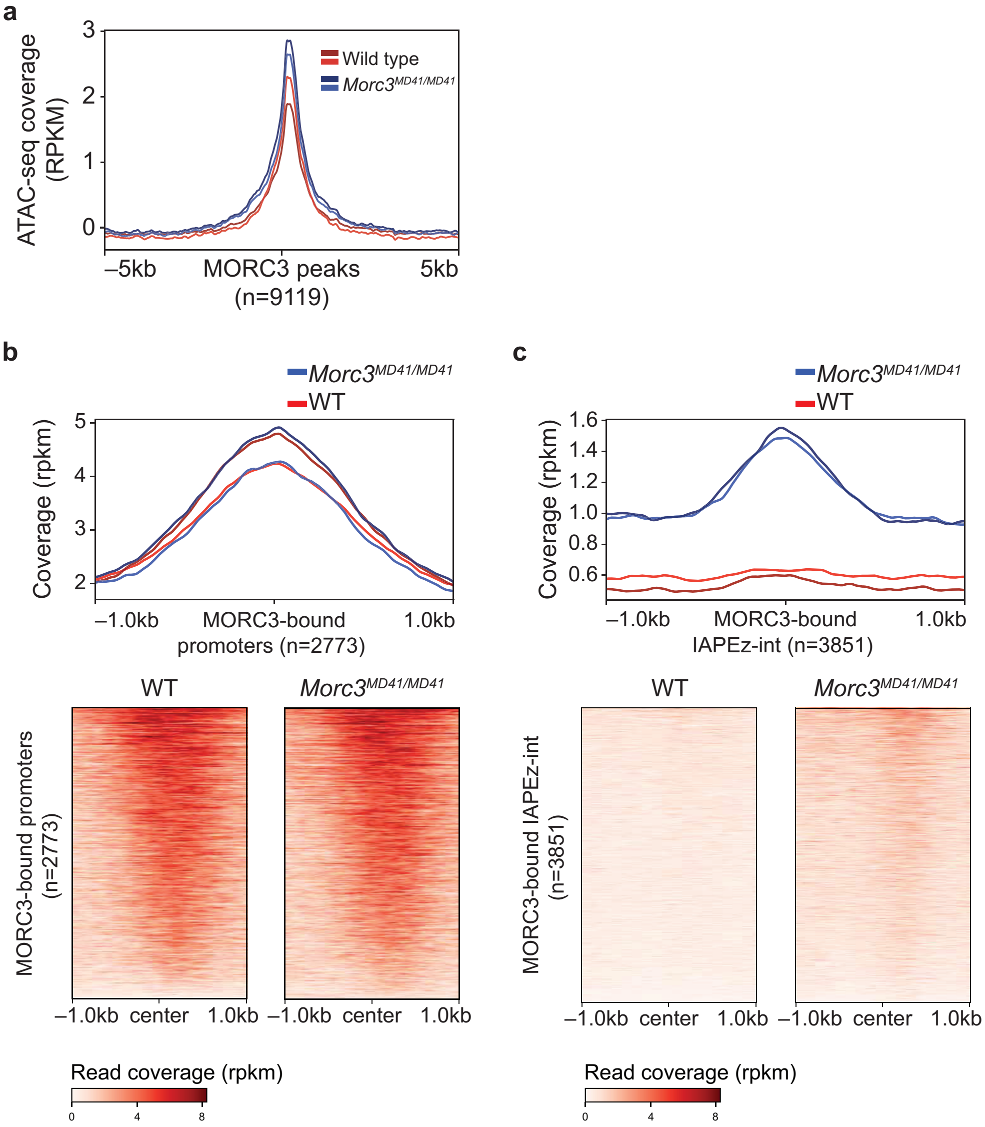

Figure S11: a Metaplot showing ATAC-seq read coverage at all MORC3 peaks. **b,c** Metaplots and heatmaps showing ATAC-seq read coverage at MORC3 bound promoters (b) and at MORC3 bound IAPEz-int (c) in WT and *Morc3*$^{MD41/MD41}$.

Figure S12:

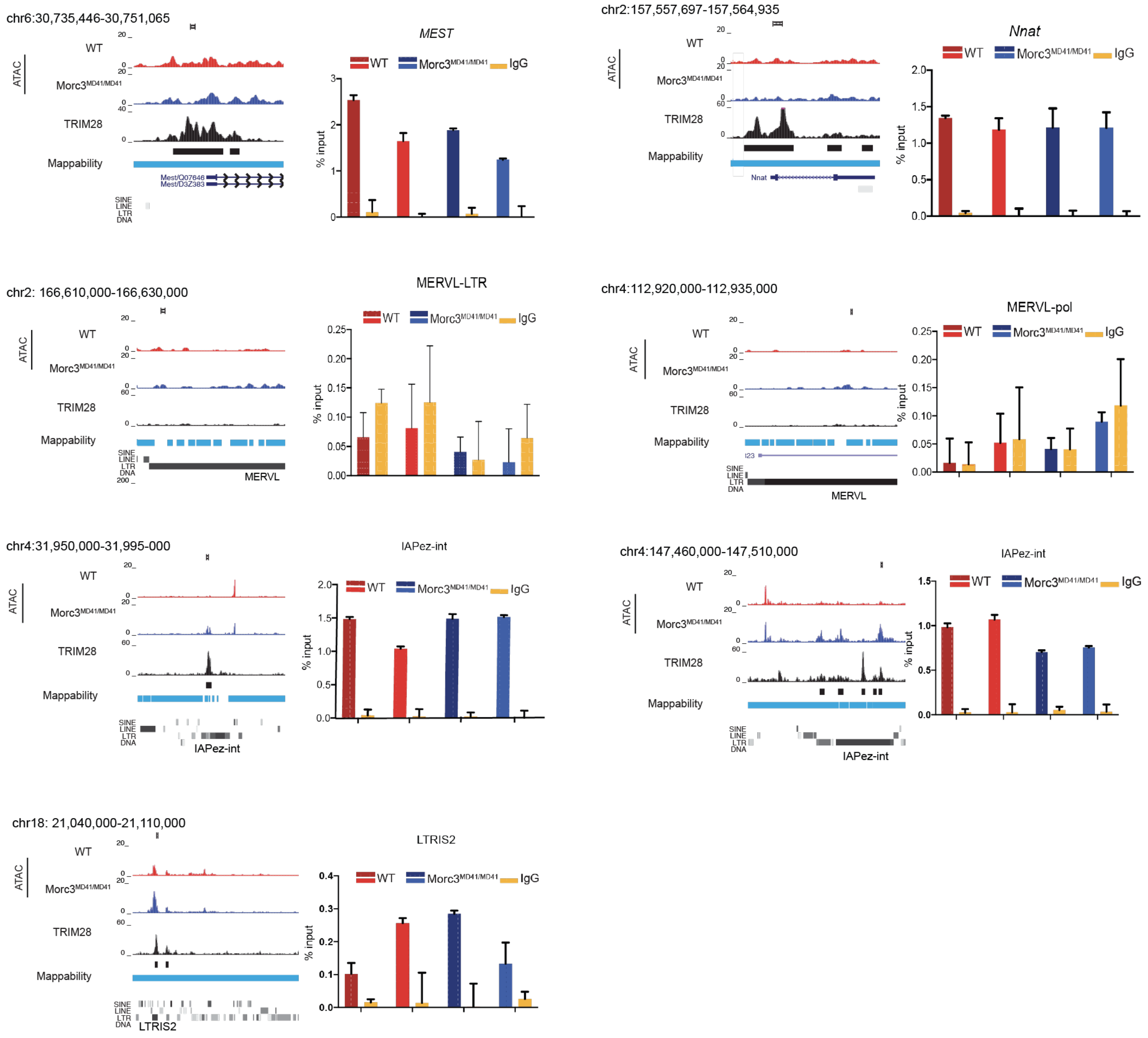

Figure S12: TRIM28 ChIP-qPCR to validate the presence of TRIM28 at the *Mest* and *Nnat*. MERVL-LTR and MERVL-pol loci were used as negative control of the TRIM28 ChIP-qPCR. TRIM28 ChIP-qPCR was performed at DALs IAPEz-int and LTRIS2.
